# Attention-deficit hyperactivity disorder and nonsuicidal self-injury in a clinical sample of adolescents: the role of comorbidities and gender

**DOI:** 10.1186/s12888-018-1620-3

**Published:** 2018-02-06

**Authors:** Judit Balázs, Dóra Győri, Lili Olga Horváth, Gergely Mészáros, Dóra Szentiványi

**Affiliations:** 1 Institute of Psychology, Eötvös Loránd University, Izabella str. 46, Budapest, 1064 Hungary; 2 Vadaskert Child and Adolescent Psychiatry Hospital, Budapest, Hungary; 3 Doctoral School of Psychology, Eötvös Loránd University, Budapest, Hungary; 4 Semmelweis University, School of Ph.D. Studies, Budapest, Hungary

**Keywords:** Attention-deficit hyperactivity disorder, ADHD, Non-suicidal self-injury, NSSI, Comorbidities, Gender, Clinical sample, Adolescents, Dimensional, Categorical approach

## Abstract

**Background:**

The aim of the present study was to investigate the possible association between attention-deficit hyperactivity disorder (ADHD) and non-suicidal self-injury (NSSI) with special focus on the role of comorbidities and gender in a clinical sample of adolescents with both a dimensional and a categorical approach to psychopathology.

**Methods:**

Using a structured interview, the Mini International Neuropsychiatric Interview Kid and a self-rated questionnaire, the Deliberate Self-Harm Inventory, the authors examined 202 inpatient adolescents (aged: 13–18 years) in the Vadaskert Child and Adolescent Psychiatric Hospital and Outpatient Clinic, Budapest, Hungary. Descriptive statistics, Mann–Whitney U test, chi-square test and mediator model were used.

**Results:**

Fifty-two adolescents met full criteria for ADHD and a further 77 showed symptoms of ADHD at the subthreshold level. From the 52 adolescents diagnosed with ADHD, 35 (67.30%) had NSSI, of whom there were significantly more girls than boys, boys: *n* = 10 (28.60%), girls: *n* = 25 (71.40%) ((χ^2^(1) = 10.643 *p* < .001 ϕ = .452). Multiple mediation analyses resulted in a moderated mediation model in which the relationship between symptoms of ADHD and the prevalence of current NSSI was fully mediated by the symptoms of comorbid conditions in both sex. Significant mediators were the symptoms of affective and psychotic disorders and suicidality in both sexes and the symptoms of alcohol abuse/dependence disorders in girls.

**Conclusions:**

ADHD symptoms are associated with an increased risk of NSSI in adolescents, especially in the case of girls. Our findings suggest that clinicians should routinely screen for the symptoms of ADHD and comorbidity, with a special focus on the symptoms of affective disorders and alcohol abuse/dependence psychotic symptoms to prevent NSSI.

## Background

Attention-deficit hyperactivity disorder (ADHD) –the main symptoms of which are inattention, hyperactivity and impulsivity (American Psychiatric Association, 2013) – is one of the most common psychiatric disorders, with a prevalence of 4-6% in children and adolescents [1, 2] and up to two-thirds of them have impairment in adulthood as well [3]. Several studies highlighted that people with ADHD have a significantly lower quality of life than people without ADHD [4–6]. More than two-thirds of ADHD cases have at least one comorbid psychiatric diagnosis, which can be both externalizing and/or internalizing disorders [7–9]. Moreover, it has recently become clear that ADHD is related to high suicidality in all age groups, both in girls and boys [10–14] and comorbid conditions mediate this association [15].

During the last decades, several clinicians and researchers suggested that besides categorical diagnosis of psychiatric disorders, dimensional approaches should be taken into account both in clinical work and research [16–20]. Recently our research group completed a systematic review on subthreshold ADHD [21]. We found that different definitions of subthreshold ADHD exist, the common point being that the definition of subthreshold ADHD does not fulfil the required criteria of the threshold ADHD definition (according to the classification systems)in children and adolescents [21]. We concluded that the prevalence rate of subthreshold ADHD is wide-ranging (0.8–23.1%), the comorbidity of subthreshold ADHD is high, up to 7% of people with subthreshold ADHD had at least one comorbid disorder, and there are several areas where subthreshold ADHD has a meaningful impact on functioning – for example, children with subthreshold ADHD had a significantly increased risk of grade retention and graduation failure, had fewer friends, more negative reputations and reported lower levels of friendship quality than children without subthreshold ADHD [21]. Moreover our previous results highlighted that even subthreshold ADHD increases the prevalence of suicidality in children and adolescents [15].

Non-suicidal self-injury (NSSI) became an individual diagnosis for the first time in the Diagnostic and Statistical Manual of Mental Disorders 5th Edition (DSM-5), under Section III. ‘Conditions for Further Study’ [22]. The definition of NSSI in DSM-5 emphasizes the deliberate, non-suicidal purpose of self-injury, which is not a culturally sanctioned behaviour. The prevalence of NSSI in an adolescent population sample was found to be 13–45% [23, 24], while it was 4% in adults [25] and 40–80% in a clinical sample of adolescents [26, 27]. It is also well known today that NSSI is often comorbid with both internalizing and externalizing disorders [28, 29]. Recently, several researchers have focused on the connection between self-injurious behaviours and suicide and found that the two phenomena often overlap [26, 30, 31], and share comorbid conditions, such as borderline personality disorder [32], depression and/or anxiety [33], and/or alcohol dependence [34], and/or ADHD (see below). However, several factors seem to distinguish suicide and NSSI, such as the frequency and methods of self-injuring and specific comorbid and personality disorders [32, 35].

Researchers have been recently a growing interest in a possible association between ADHD and self-injury, including NSSI – already two systematic review papers have summarized the results and found a strong association between ADHD and self-injury [28, 29]. One of the common traits of the two phenomena is poor response inhibition, which is associated with impulsivity [36, 37]. A further question is whether there is a direct association between the symptoms of ADHD and the prevalence of NSSI or whether comorbid conditions mediate this association. Swanson et al. [38] found, in a sample of girls with ADHD, aged 6–12, that the association between ADHD and NSSI is mediated by impulsivity and other symptoms of externalizing disorders. Taylor et al. [39] found, in an adult normal population, that there are significant associations between ADHD symptom severity and self-injury and suicidal behaviour (including ideation and attempts) and they are mediated by comorbidities, such as affective, anxiety, drug, and alcohol abuse disorders and emotion-focused coping style. Based on a 10-year follow-up study of girls aged 6–12, with and without ADHD, and the onset of self-injury in their adulthood, Meza et al. [37] suggested that childhood response inhibition predicted young-adult suicidality, including ideation, suicide attempts and NSSI, and peer victimization in adolescence emerged as a significant partial mediator of young-adult response inhibition and NSSI linkage.

According to our knowledge, there is no previous study that has investigated,in both adolescent girls and boys, whether there is a direct association between their symptoms of ADHD and the prevalence of NSSI or whether comorbidity between ADHD and NSSI conditions mediate this association. Therefore, the objective of the current cross-sectional study was to investigate the prevalence of NSSI in an inpatient clinical sample of adolescent girls and boys, either with the symptoms or with a full diagnosis of ADHD. Although, the cross-sectional nature of the analyses preclude causal inferences, we planned to examine whether there is a direct association between ADHD symptoms (fulfilling or not the diagnostic threshold) and the prevalence of NSSI and how the symptoms of comorbid psychiatric conditions influence this, and whether there is a difference between girls and boys at this age.

## Methods

### Ethics

The Ethical Committee of the Medical Research Council, Hungary (ETT-TUKEB) approved the study. The parents of all adolescents included in the study, and adolescents older than 14 years provided written informed consent after the nature of the study had been explained. There was no economic or any other type of compensation provided to the participants.

### Subjects

Adolescents, who were inpatients in the Vadaskert Child and Adolescent Psychiatric Hospital and Outpatient Clinic, Budapest, Hungary between 25.02.2015 and 09.05.2016, were included in the study. Inclusion criteria were that the adolescent had to be aged between 13 and 18 years and he/she had to be psychiatric inpatient. Exclusion criterion was mental retardation in the medical history.

### Measures

Psychiatric symptoms and disorders with both subthreshold and fulfilling diagnostic criteria of the classification systems were evaluated by the Hungarian version of the modified Mini International Neuropsychiatric Interview Kid (M.I.N.I. Kid) 2.0 [40–43]). The M.I.N.I. Kid is a structured diagnostic interview for the assessment of major child/adolescent psychiatric disorders, such as affective disorders (i.e. major depressive episode, dysthymic disorder, hypo/manic episode, anxiety disorders (i.e. panic disorder, agoraphobia, separation anxiety disorder, social anxiety disorder, specific phobia, post-traumatic stress disorder (PTSD), generalized anxiety disorder (GAD)), obsessive-compulsive disorder (OCD), substance-related dependence/abuse (e.g. alcohol abuse/dependence and psychoactive substance abuse/dependence), Tourette’s disorder, ADHD, Conduct Disorder (CD), oppositional defiant disorder (ODD), eating disorders (e.g. anorexia nervosa, bulimia nervosa), adjustment disorder, psychotic disorders and suicidality. The interviewer posed the questions of the M.I.N.I. Kid to the adolescent. In its original form, the structure of the M.I.N.I. Kid is branching, which means, if the core symptoms of a disorder are not present, the additional questions on the symptoms of that disorder should not be asked. However, in the current study we used the modified version of the M.I.N.I. Kid, which means we excluded the ‘branching logic’ and in this way the M.I.N.I. Kid evaluated all the possible symptoms of a disorder. To ensure inter-rater reliability, all interviewers participated in a training course before the study and during the study interviewers were regularly supervised.

Self-injuries were evaluated by the Deliberate Self-Harm Inventory (DSHI) [44]. DSHI is a self-rated questionnaire, which assesses whether people engage in direct self-injury behaviour, self-inflicted damage of the surface of an individual’s body by self-cutting, self-burning, self-hitting, self-biting and skin damage by other methods. The questionnaire contains 17 items. The questions on the method of self-injury should be answered by a ‘yes’ or ‘no’. DSHI comprises facets on frequency, severity and duration of self-injury. The DSHI is branching, which means, if all questions on the method of self-injury are answered with ‘no’, the additional questions should not be asked. However, if there is at least one ‘yes’ answer, then the following questions on the age onset, frequency, duration and severity of self-injury should be asked.

### Diagnostic definitions

The definition of psychiatric disorders, including ADHD and all comorbid diagnoses, were based on DSM-IV [45]. The definition of subthreshold ADHD required the presence of more than five symptoms of ADHD [15].

Current suicidality was defined as having any suicidal ideations and/or suicide attempt with direct desire to end one’s life currently and/or in the past month. The presence of current suicidality was noted if the child answered ‘yes’ to any of the following questions: In the past month did you: Wish you were dead?;, Want to hurt yourself?; Think about killing yourself?; Think of a way to kill yourself?; Attempt suicide? [40–43].

NSSI was defined according to DSM-5 [22] as a: deliberate, non-suicidal purpose of the self-injurious act, which is not socially sanctioned. It is important to distinguish it from drug overdoses, culturally sanctioned behaviours (e.g. piercings), and repetitive, stereotypical forms among people with developmental disorders. Self-injurious acts should have occurred on at least 5 occasionsin the past year. Moreover, the individual who engages in NSSI must have the aim of achieving a better emotional state after the action [22].

### Statistics

Data were analysed using IBM SPSS Statistics 22.0. Descriptive statistics, Mann–Whitney U test, Chi-square test and mediator model were used. In our survey we used bootstrapping procedure (5000 bootstrap sample) in mediator model. Mann-Whitney U test was used in order to show the age difference between gender, and Chi-square test was used to analyze gender differences among adolescents with both ADHD and NSSI. Those M.I.N.I. Kid [40–43] diagnoses that were comorbid with ADHD and had a prevalence of3% or more, were included in comparative and mediator model analyses. According to this the following diagnoses were not included in the statistical analyses: Tourette’s syndrome, tic disorder, anorexia nervosa, bulimia nervosa, adjustment disorder, autism spectrum disorder.

In the case of Chi-square and Mann–Whitney U test analyses, the data of those adolescents who had a diagnosis of ADHD according to the M.I.N.I. Kid were included. The data of those adolescents who had no ADHD diagnosis but who had other diagnoses were excluded.

While dichotomous variables cannot be used in a mediator model and only 10 variables can be handled in the model, we added the number of symptoms of the M.I.N.I. Kid diagnoses, and these continuous variables were involved in the model. Thus, in the case of the mediator model we enrolled the whole group of adolescents (*n* = 202): both those who had an ADHD diagnosis according to M.I.N.I. Kid and those who did not have an ADHD diagnosis, but had more than five ADHD symptoms (subthreshold ADHD), and also those who had fewer than five ADHD symptoms. In the mediator model, the independent variable was the amount of ADHD symptoms and the outcome variable was the number of self-harm-forms according to the DSHI [40], and gender was included as covariate. NSSI variable is based on sum of all 17 Deliberate Self-Harm Inventory (DSHI) items. It means that this variable is created as a sum of YES answers of occurred types of self-harming behavior, because many individuals who self injure use more than one method [44]. In order to show the gender differences, we used gender as the moderator variable in the moderated mediator model.

Mediator variables were the following grouped diagnoses: a) affective disorders: major depressive episode, dysthymic disorder, hypo/manic episode; b) anxiety disorders: panic disorder, agoraphobia, separation anxiety disorder, social anxiety disorder, specific phobia, PTSD, GAD; c) OCD; d) CD and ODD; e) alcohol abuse and dependence; f) psychoactive substance abuse and dependence; g) psychotic disorder; h) suicidality.

The study is asserting statistical mediation with cross-sectional data.

## Results

### Subjects

The study population included 202 psychiatric inpatient adolescents, 99 (49%) boys and 103 (51%) girls. Mean age of the whole study population was 14.87 years (SD = 1.38), the mean age of boys was 14.8 years (SD = 1.43), while the mean age of girls was 14.94 years (SD = 1.35). There was no significant difference between the mean age of boys and girls (U = 4771.000 z = −.806 *p* > .05).

Altogether 52 (25.7%) adolescents fulfilled the diagnosis of ADHD according to M.I.N.I. Kid, 23 (44.2%) boys and 29 (55.8%) girls. The mean age of adolescents diagnosed with ADHD was 14.75 years (SD = 1.25). The mean age of boys with ADHD was 14.95 years (SD = 1.29), and the mean age of girls was 14.58 years (SD = 1.21). There was no significant difference between the mean age of boys and girls diagnosed with ADHD (U = 290.000 z = −.832 *p* > .05).

Additionally, there were 77 (38.1%) adolescents who had more than five ADHD symptoms – which was the threshold for subthreshold ADHD – but less than the required criteria according to the DSM-IV. The mean age of these adolescents was 14.88 years (SD = 1.53). The 77 adolescents with subthreshold ADHD included 40 boys (52%) and 37 girls (48%). The mean age of boys was 15 years (SD = 1.55), and of girls it was 14.76 years (SD = 1.52). There was no significant difference between the mean age of boys and girls with subthreshold ADHD (U = 675.500 z = −.673 *p* > .05).

Of the 52 adolescents diagnosed with ADHD, 35 (67.30%) had NSSI – 10 (28.60%) boys and 25 (71.40%) girls. There were significantly more girls than boys among the adolescents ‘with both ADHD and NSSI(χ^2^(1) = 10.643 *p* < .001 ϕ = .452).

Table 1 presents the prevalence of the most common comorbid mental disorders of adolescents with ADHD and NSSI (*n* = 35).

**Table 1:** The prevalence of the most common comorbid mental disorders of adolescents diagnosed with ADHD and NSSI (*n* = 35)

Mental disorders	Prevalence %	n
Suicidality	94	33
ODD	66	23
GAD	63	22
Psychotic disorder	60	21
Mania	51	18
Social anxiety disorder	49	17
OCD	46	16
Major Depressive Episode	43	15
Dysthymia	34	12
Panic disorder	31	11
Separation anxiety disorder	26	9
CD	23	8
Agoraphobia	23	8
Psychoactive substance dependence	23	8
Hypomania	20	7
Specific phobia	20	7
Alcohol abuse /dependence	20	7
Psychoactive substance abuse	20	7
PTSD	17	6

*CD* Conduct Disorder, *GAD* Generalized Anxiety Disorder, *OCD* Obsessive Compulsive Disorder, *ODD* Oppositional Defiant Disorder, *PTSD* Posttraumatic Stress Disorder

**Fig. 1:**
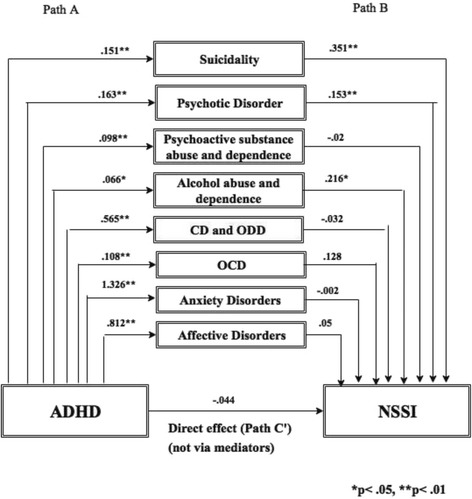
Mediation model including path A, path B and the direct pathway/effect between ADHD and NSSI (C′). Path A: The effect of the symptoms of ADHD on comorbid mental disorders. Path B: The effect of the comorbid mental disorders on prevalence of NSSI. CD: Conduct Disorder, OCD: Obsessive Compulsive Disorder, ODD: Oppositional Defiant Disorder

Figure 1. shows the mediation model including path A and path B and the direct pathway/effect between ADHD and NSSI (C′). Path A presents the effect of the symptoms of ADHD (independent variable) on comorbid mental disorders (mediating factors). The symptoms of ADHD predict significantly the symptoms of all measured comorbid mental disorders. Path B presents the effect of comorbid mental disorders on the prevalence of NSSI. Alcohol abuse and dependence, psychotic disorder, suicidal behaviour significantly predict the appearance of NSSI. Gender was included as covariate.

**Fig. 2:**
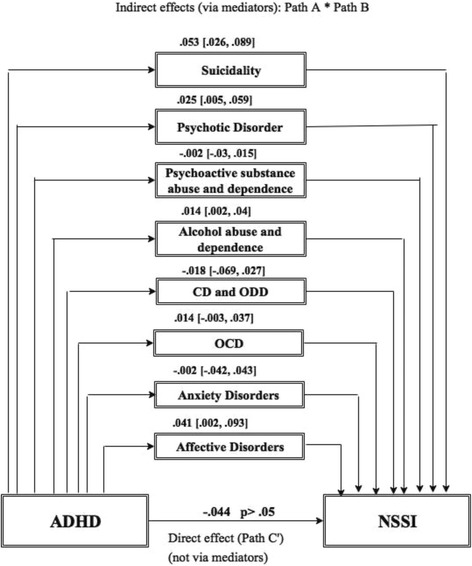
Mediation model including the indirect pathway/effect via mediating factors (Path A* Path B), and the direct pathway/effect (C′) between ADHD and NSSI. *CD:* Conduct Disorder, OCD: Obsessive Compulsive Disorder, ODD: Oppositional Defiant Disorder. Bootstrap-method, BootLLCI-BootULCI: 95% confidence interval: confidence interval not containing 0 implies that there is statistically significant effect

Figure 2. shows the indirect pathway/effect between ADHD and NSSI via mediating factors (path A* path B). The linkage between ADHD and NSSI was fully mediated by comorbid mental disorders. Mediating factors were affective disorders, alcohol abuse and dependence, psychotic disorder and suicidal behaviour. There was no evidence of a statistically significant direct pathway (direct effect (C′) from the symptoms of ADHD to the prevalence of NSSI.

**Fig. 3:**
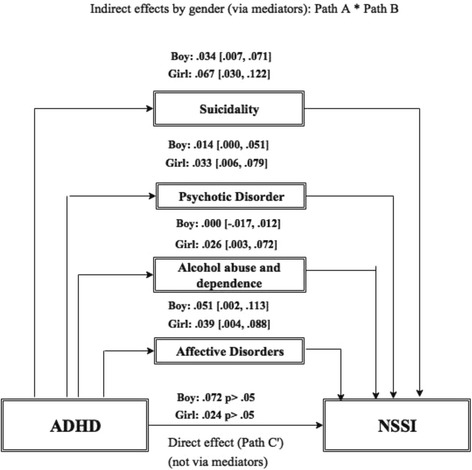
Mediation model including the significant indirect pathway/effect via mediating factors (Path A* Path B), and the direct pathway/effect between ADHD and NSSI (C′) by gender. Bootstrap-method, BootLLCI-BootULCI: 95% confidence interval: confidence interval not containing 0 implies that there is statistically significant effect

The association among ADHD, NSSI and comorbid mental disorders was analysed with Spearman correlation separately for boys and girls (*N* = 202). According to this result moderated mediation model contains direct and the significant indirect effects by gender (Fig. 3). There was no evidence of a statistically significant direct pathway from the symptoms of ADHD to NSSI either in boys or in girls. The association between the symptoms of ADHD and the prevalence of NSSI was mediated – indirect effect – by comorbid mental disorders. Mediating factors were for both genders: affective disorders, psychotic disorder, and suicidal behaviour. In addition to these, alcohol abuse and dependence were mediating factors only for girls.

## Discussion

Although there has recently been a growing interest in a possible association between ADHD and NSSI [29, 46], to the best of our knowledge this is the first study to have investigated, in an adolescent clinical sample including both girls and boys, whether there is a direct effect of the symptoms of ADHD on NSSI or ‘whether comorbidities mediate between these conditions and whether there is an effect of gender on this association.

Although the main focus of the current study was not on the prevalence data, first of all we would like to highlight that based on the self-rated screening tool, more than two-thirds of the adolescents with ADHD had NSSI. Similar to earlier findings [23, 47, 48], in our ADHD subsample gender had a significant effect on NSSI: we found that more than two-thirds of the adolescents with ADHD and NSSI were girls, that is to say that less than half of the boys and more than 85% of the girls had NSSI. Later, we will come back to the discussion of the gender differences found in ADHD and NSSI. All these results have high clinical significance: clinicians should screen for the risk factors of NSSI in their patients with ADHD, with a special focus on girls.

Our results are in line with those previous studies that stated that people with ADHD have a higher risk than those without of developing comorbid psychiatric problems, both externalizing and internalizing ones [7–9]. Focusing on comorbidity of those adolescents with ADHD, who had NSSI as well, in the current study the most common comorbidity next to ADHD and NSSI was suicidality followed by the symptoms of ODD, GAD and psychotic disorders, all of which were present in more the two-thirds of patients with ADHD and NSSI. Almost half of the patients with ADHD and NSSI had comorbid symptoms of manic episode, social phobia and OCD, and one-third of them had the symptoms of comorbid alcohol abuse /dependence, dysthymic and panic disorder, while one-quarter of them had the symptoms of comorbid separation anxiety, agoraphobia, conduct or substance use disorder.

Coming to the discussion of the main focus of the current study, we found that there is no direct association between the symptoms of ADHD and the prevalence of NSSI in a clinical sample of adolescents in any sex. Comorbidities fully mediate the association between these conditions. In both sexes the symptoms of affective disorders, suicidality and psychotic disorders were significant mediators between the symptoms of ADHD and NSSI, while in the case of girls so too were the symptoms of comorbid alcohol abuse/dependence.

There is increasing evidence that NSSI is most often used to attempt to avoid, regulate, reduce or escape from negative emotions, such as anxiety, sadness, or guilt [27, 28, 49]. Even the criterion of NSSI in DSM-5 includes that the individual who engages in NSSI must have the aim of achieving a better emotional state after the action [22]. Moreover the symptoms of ADHD have several negative impacts in everyday life, such as the adolescent cannot pay attention during task sat school or in homework, often has conflict with parents, teachers and peers due to hyperactivity and impulsivity, again the DSM-5 definition includes this criterion as well [22]. All these can all lead to low self-esteem, frustration and depressed mood. This mechanism may explain the mediator role of the symptoms of depression between the symptoms of ADHD and NSSI.

We would like to draw attention to the fact that based on this ADHD inpatient adolescent study population our result supports those recent studies that suggested a strong connection between self-injurious behaviours and suicide [5, 30, 31]: almost all (94%) of our inpatient adolescents with ADHD and NSSI had comorbid suicidality.

Interestingly, an internationally new result in this study is the high comorbidity of the symptoms of psychotic disorder with ADHD and NSSI. The reason for this high psychotic symptom comorbidity may be that the population of the current study included inpatients with ADHD, who may have been admitted to a psychiatric unit not only – or possibly often not – due to the consequences of ADHD, but also because of the presence of severe comorbidities, such as psychotic symptoms. A possible explanation for this new result could be that what Hafner et al. [50] found based on their overview from 1996 to 2016, comparing people with schizophrenia who were in the prodrome at first admission to those with unipolar depression and with healthy controls and then analysed the medium-term (5-year) and long-term (12-year) data. They found that the prodromal stages of schizophrenia and depression were very similar until positive symptoms appeared. The most frequent symptom in schizophrenia is depressed mood [50].

Our results are consistent with those studies that found high comorbidity of NSSI and alcohol abuse/dependence [51] and ADHD and alcohol abuse/dependence [52]. Moreover, Fulwiler et al. [51] did not find differences in the prevalence of alcohol abuse/dependence in the case of people with self-injurious and suicidal behaviour, which could be another explanation of our result on the connection of self-injurious behaviours and suicidality, as described above. Lam et al. [53] also suggest that alcohol and other substance abuse/dependence comorbidity of ADHD could be the link between self-injury and suicidality. Izutsu et al. (2006), focusing on adolescents, found that the prevalence of childhood ADHD is higher among those adolescents with self-injury behaviour, who had comorbid substance use. Furthermore, Hurtig et al. [33] found in their longitudinal cohort study that substance abuse/dependence comorbidity, including alcohol, significantly predicts later NSSI (next to CD and family conflicts) in patients with ADHD.

Previous results on the influence of sex on the pattern and course of comorbid psychopathological conditions ‘concurrent with ADHD are still controversial [54–58]. In the current study, besides with the symptoms of psychotic disorders, we found gender differences – again a higher prevalence of girls than boys – in the case of alcohol abuse/dependence and major depressive episode in the comorbidity of ADHD and NSSI, however the differences were only tendencies. One-third of girls with ADHD and NSSI had alcohol dependence as well, while only 10 % of the boys with ADHD and NSSI had this comorbidity. Our result supports those previous researches that found that girls with ADHD have a higher risk of having substance use, including alcohol, than boys [59–63]. The higher prevalence of the comorbidity of psychotic symptoms with ADHD and NSSI in girls needs further research, while previous data supported that in the general population the lifetime prevalence of psychotic symptoms is equal in girls and boys, however the onset of this disorder is earlier in boys [64]. Moreover, examining that part of our ADHD subsample who had no NSSI, we did not find differences in the comorbidity between girls and boys, only that there was a tendency in the case of separation anxiety disorder (again it was higher in girls than in boys). This result supports those previous studies that found that gender does not have an effect on the prevalence of comorbidity with ADHD [65, 66].

Limitations of these findings include their being cross-sectional, therefore no causal relationship was revealed among the investigated factors, we cannot ensure that the mediator (i.e., comorbid diagnoses/symptoms) came after ADHD symptoms and before the NSSI, moreover mediator variables may temporally occur in between the predictors and the criterion measures. Our data are based self-reported NSSI, which can be biased, i.e., it can be an underestimation, why adolescents may want to deny it. Moreover, we could not use age at which NSSI first occurred in our analyses because there were too many missing and inappropriate data among answers related to this question. Finally, as described the study population are inpatients with ADHD, which suggests that these patients belonged to the more severe end of the spectrum, and in addition the reason for hospitalization could have been comorbid diagnoses, which can be severe comorbidities as well.

## Conclusions

In conclusion, the high prevalence of NSSI, especially in the case of girls, and its overlap with suicidality among adolescents with the symptoms of ADHD, calls to the attention of clinicians the importance of screening routinely for the risk factors of NSSI and suicidality in this population, with a special focus on girls. While the symptoms of comorbid conditions of ADHD, such as affective disorders, suicidality, alcohol abuse/dependence and psychotic disorders, fully mediate the relation between the symptoms of ADHD and NSSI, the early recognition and treatment of the symptoms of ADHD and these conditions can be important in the prevention of NSSI.

## Abbreviations

ADHD: Attention-Deficit Hyperactivity Disorder
CD: Conduct Disorder
DSHI: Deliberate Self-Harm Inventory
DSM-5: Diagnostic and Statistical Manual of Mental Disorders 5th Edition
ETT-TUKEB: Ethical Committee of the Medical Research Council, Hungary
GAD: Generalized Anxiety Disorder
M.I.N.I. Kid: Mini International Neuropsychiatric Interview Kid
NSSI: Non-Suicidal Self-Injury
OCD: Obsessive-Compulsive Disorder
ODD: Oppositional Defiant Disorder
PTSD: Post-Traumatic Stress Disorder
